# Finite element modeling of the viscoelastic responses of the eye during microvolumetric changes

**DOI:** 10.4236/jbise.2013.612A005

**Published:** 2013-11-01

**Authors:** Benjamin Cruz Perez, Hugh J. Morris, Richard T. Hart, Jun Liu

**Affiliations:** 1 Department of Biomedical Engineering, The Ohio State University, Columbus, USA; 2 Department of Ophthalmology, The Ohio State University, Columbus, USA

**Keywords:** Viscoelasticity, Finite Element Modeling, Intraocular Pressure, Cornea, Sclera

## Abstract

A linear viscoelastic finite element model was built to investigate factors that influenced the intraocular pressure (IOP) elevations due to micro-volumetric changes in the eye at three different rates. The viscoelastic properties of the cornea and the sclera, including the instantaneous modulus, equilibrium modulus, and relaxation time constants, parametrically varied to examine their effects on IOP elevations at different rates of volumetric changes. The simulated responses were in good agreement with the previously reported experimental results obtained from porcine globes, showing the general trend of higher IOP elevations at faster rates. The simulations showed that all viscoelastic properties influenced the profile of the dynamic IOP due to volumetric changes, and the relative significance of a specific parameter was highly dependent on the rate of change.

## 1. INTRODUCTION

Glaucoma is one of the leading causes of blindness worldwide [1]. It is known to be associated with elevated intraocular pressure (IOP), although the mechanisms of IOP-induced optic nerve damages are not well understood. The longstanding focus in the glaucoma field has been on the steady-state IOP [2], but it is also known that the IOP fluctuates during the day [3], as well as during normal physiological activities such as postural change [4,5], valsalva maneuver [6,7], water drinking [8], and eye movement [9]. Previous studies have shown that these short-term IOP fluctuations may be linked to glaucoma risk [8,10,11].

Because it has been very difficult to characterize the IOP-associated mechanical effects on ocular structures *in vivo*, computational modeling has been a helpful tool to describe and predict the biomechanical responses to IOP. Using finite element modeling of the posterior portion of the eye, Sigal *et al*. found that the material properties (*i.e*., the elastic modulus) of the peripapilary sclera played an important role in determining the deformation and stress states of the optic nerve head (ONH) [12], which was the primary site for early glaucomatous damages.

These findings have led to a growing interest in studying the biomechanical properties of the ocular shell and the association with glaucoma progression. Characterization of the mechanical properties of the cornea and sclera has been conducted, including uniaxial [13–16], biaxial [17], and inflation testing [18–22]. These studies showed that cornea and sclera exhibit the typical nonlinear, anisotropic, and viscoelastic mechanical behavior seen in many other biological tissues [23–27].

Very little has been done to understand the response of the eye to the short-term changes of volume and pressure. Recently, our group examined IOP elevations in porcine globes due to short-term micro-volumetric infusions. We found a strong rate-dependent response showing that a fast volumetric change induced a significantly higher IOP change in the same eye compared to slow volumetric changes [28]. The current study aims to build a finite element model of the whole corneoscleral shell (omitting the optic nerve head) to investigate how cornea and sclera viscoelastic properties influence the eye’s response to microvolumetric changes and how each factor contributes to the rate-dependent response.

The whole eye models have been developed in the past for understanding ocular physiology. Srodka [29] utilized an axisymmetric finite element of the whole eye model to evaluate the accuracy of the standard clinical method for IOP measurement, the Goldmann applanation tonometry. Anderson *et al*. [30] utilized a 3D hyperelastic whole eye model to investigate the influence of the boundary conditions at the cornea and sclera conjunction on the apical rise of the cornea during inflation. Our current study incorporated the general formulation of these previous whole eye models such as using an axisymmetric geometry and omitting the ONH.

## 2. METHODS

### 2.1. Finite Element Modeling

An axisymmetric model of the corneoscleral shell of a porcine eye was constructed in COMSOL (v4.3, Burlington, MA, USA) using a series of ellipses assuming symmetry along the optic axis. The ellipsoidal building blocks allowed for realistic representation of the thickness variations in the corneoscleral shell. The central cornea thickness (CCT) was set to 0.96 mm [31], with a radius of curvature of 7.5 mm, an assumed eccentricity of 0.5, and a white-to-white (WTW) diameter of 13.5 mm. The scleral radius of curvature was set to 12.8 mm, the anterior thickness to 0.89 mm, the equatorial thickness to 0.58 mm, and the posterior thickness to 1.12 mm. Because of the oblate spheroidal shape of the porcine sclera, the ratio between the posterior radius and the equatorial radius was set to 0.86. These dimensions were the average measurements in porcine eyes (SiouxPreme Packing Co, Sioux City, IA) acquired for other studies in our laboratory.

**Figure 1:**
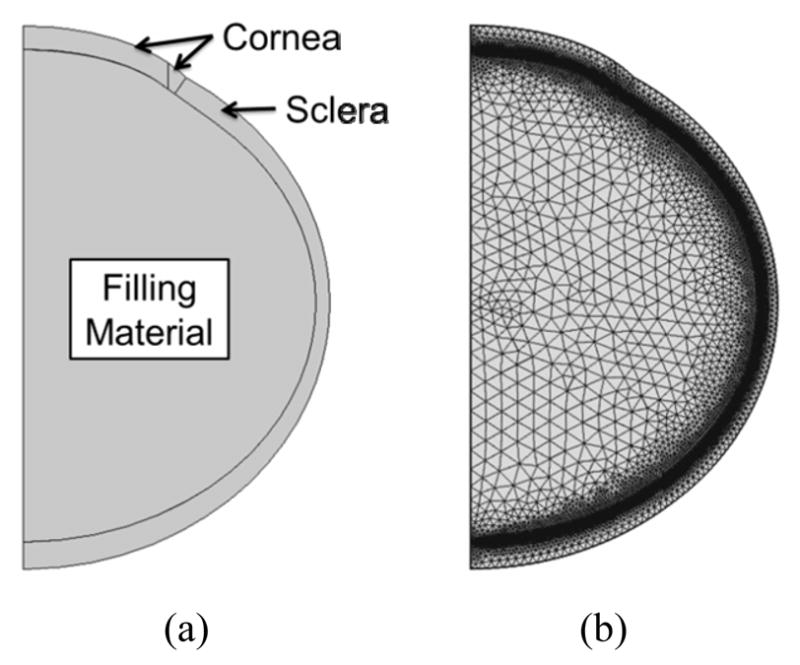
(a) General geometry of the porcine eye; and (b) Typical finite element mesh.

The cornea and sclera were divided by a radial line going through the center of the sclera ellipse. A small transition zone at the cornea-sclera junction was implemented with a finer mesh to avoid convergence difficultties at the boundary of material property change. A filling material was used to fill the inner space of the ocular shell and to couple the intraocular volumetric change to the mechanical responses of the shell (as described subsequently). Figure 1(a) shows the constructed geometry of the whole eye as described above, and Figure 1(b) illustrates the typical finite element mesh.

The mesh was constructed using quadratic triangular elements. A displacement mesh convergence test was performed with five different mesh densities that monitored five different locations to ensure mesh accuracy. The mesh density chosen for this study had approximately 11,900 elements for a total of 54,000 degrees of freedom (Figure 1(b)). A time-dependent solver in COMSOL was utilized for solving the constitutive equations.

### 2.2. Simulated Infusions

Our laboratory has previously reported an experimental infusion study on porcine whole globes at three different rates (the details of the experiments are provided in the referenced article) [28]. In the present study, the simulations were performed following the design of the infusion experiments. Specifically, three different infusion rates (as shown in Table 1) were implemented to represent the typical time scales seen in short-term IOP fluctuations (seconds to minutes) [28]. The total volume change was set to 15 μL.

### 2.3. Using Thermal Expansion to Simulate Intraocular Volumetric Change

In order to simulate intraocular volume increments, it was computationally convenient to use a thermal expansion model although in reality there is no temperature dependence being studied. However, by assuming that the tissue and fluids inside the ocular shell are incomepressible, the volume change can be modeled as a simple uniform volumetric thermal expansion of an intraocular filling material, which can be implemented in the COMSOL software to conveniently provide mechanical coupling between the shell and the intraocular material. The thermal expansion model assumed that all mesh points within the enclosed filling material had a heat source and the total power (*P*) was computed by the following relation:

(1) $$P=\frac{dV}{dt}\frac{C_{p}\rho }{\alpha_{V}}$$

where d*V/*d*t* is the volumetric change rate, *C_p_* is the specific heat, *ρ* is the density and *α_V_* is the coefficient of thermal expansion for the filling material. The parameters for low density polyethylene (LDPE) was used to simulate the filling material because of its high coefficient of thermal expansion (*α_V_* = 4 × 10^−4^ 1/°C) [32]. The volumetric change rate (d*V/*d*t*) resulted from the thermal expansion was set equal to the experimental infusion rate assuming no leakage. A fixed point was placed at the center of the filling material to ensure that it expands equally in all directions. The filling material was thermally insulated at the outer boundary to ensure conservation of energy.

### 2.4. Constitutive Model

The cornea and sclera were modeled using a nearly incompressible linear viscoelastic generalized Maxwell model. The stress-strain relationship is defined as follows [32]:

(2) $$\sigma (t)=E_{\mathrm{equ}}\cdot \varepsilon (t)+\sum_{i=1}^{m}E_{i}\cdot \dot{\varepsilon }\cdot \tau_{i}\cdot \left(1-e^{-\frac{t}{\tau_{i}}}\right)$$

where *E_equ_* is the equilibrium modulus, *E_i_* is the relaxation modulus of the *i-*th branch, *τ_i_* is the time constant of the *i-*th branch, *ε* is the strain, and *ε̇* is the strain rate. A two-branch model (*i.e*., *m* = 2) was adopted [16]. The time constants (*τ*_1_ and *τ*_2_) are denoted as the short term time constant (*τ_s_*) and the long term time constant (*τ_l_*) in the later discussions for clear differentiation. The instanttaneous (*E_inst_*) is defined as [14]:

(3) $$E_{\mathrm{inst}}={\frac{\sigma }{\varepsilon }|}_{t=0}.$$

The branch relaxation moduli were set to be equal (*i.e*., *E*_1_ = *E*_2_). Therefore, the branch relaxation moduli can be found as:

(4) $$E_{1}=E_{2}=\frac{E_{\mathrm{inst}}-E_{\mathrm{equ}}}{2}.$$

The instantaneous and equilibration moduli of the sclera were set to be 5 times of those of the cornea to ensure a stiffer response of the sclera commonly reported in experimental results and simulation studies [28,34,35]. All other parameters were allowed to vary independently.

### 2.5. Parametric Study

The values of the material parameters of ocular shell were varied within a range of the plausible values for the porcine eye (as shown in Table 2) to evaluate the effects that each parameter has on the short-term IOP elevations. To the best of our knowledge, experimental measurements of the viscoelastic properties of the porcine cornea and sclera including the instantaneous modulus, equilibrium modulus, and time constants have not been reported. The selected baseline values were estimated from approximating the model predictions with the average results obtained from the infusion experiments in porcine eyes [28].

## 3. RESULTS

**Figure 2:**
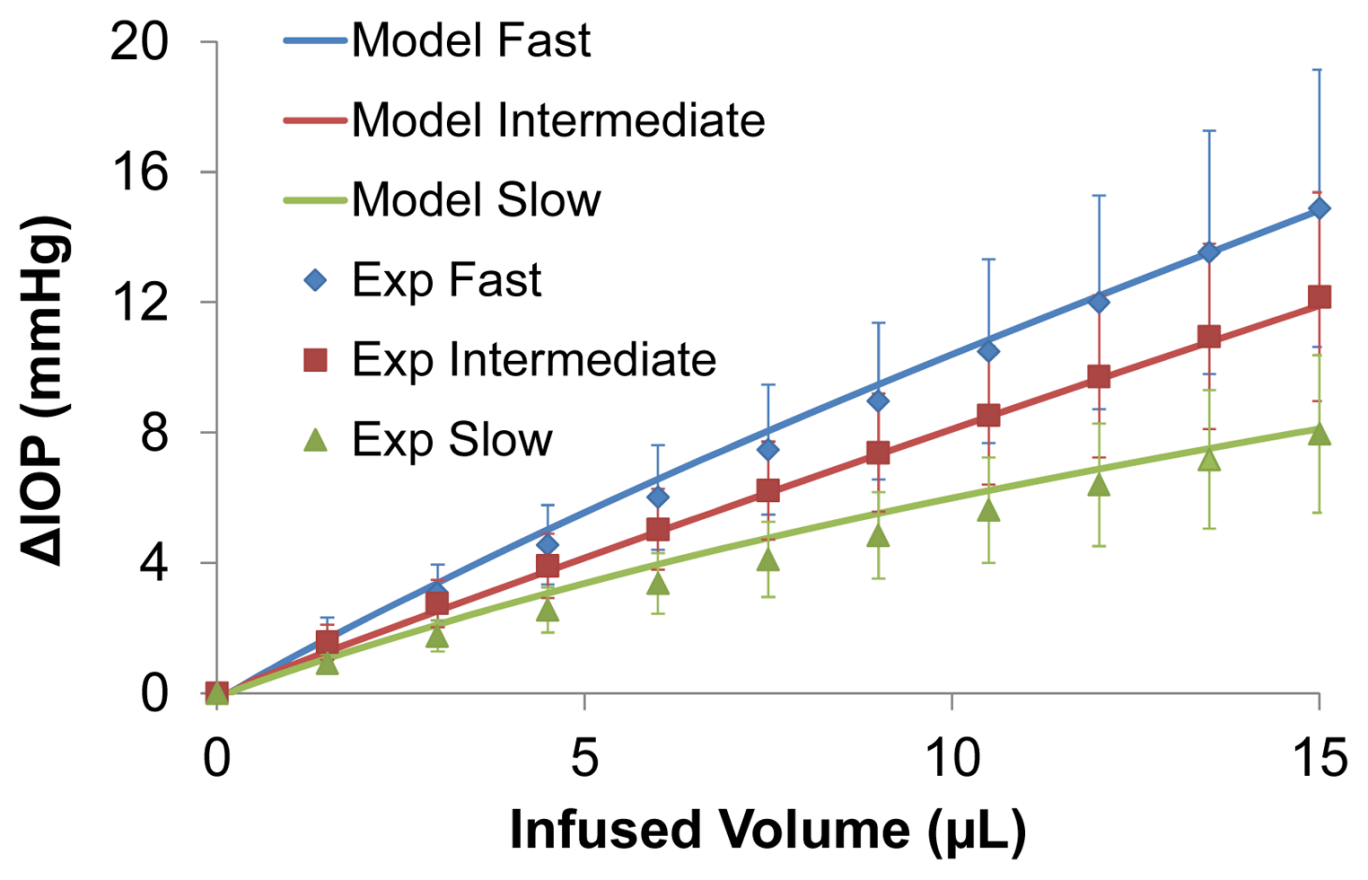
The IOP elevations (ΔIOP) as a function of the infused volume as predicted by the model (solid lines) or obtained from the experiments (markers).

A comparison of the simulated IOP/infusion volume response (solid lines) and the average experimental responses in the porcine eyes (individual markers) is presented in Figure 2. All viscoelastic properties were set at the baseline values. Selected volume levels (11 levels evenly distributed between 0 to 15 μL) were used for plotting the experimental response for visual clarity. Most of the markers fell on the corresponding curves, suggesting a good agreement between the model simulation and the experimental results could be simultaneously achieved at all three rates.

**Figure 3:**
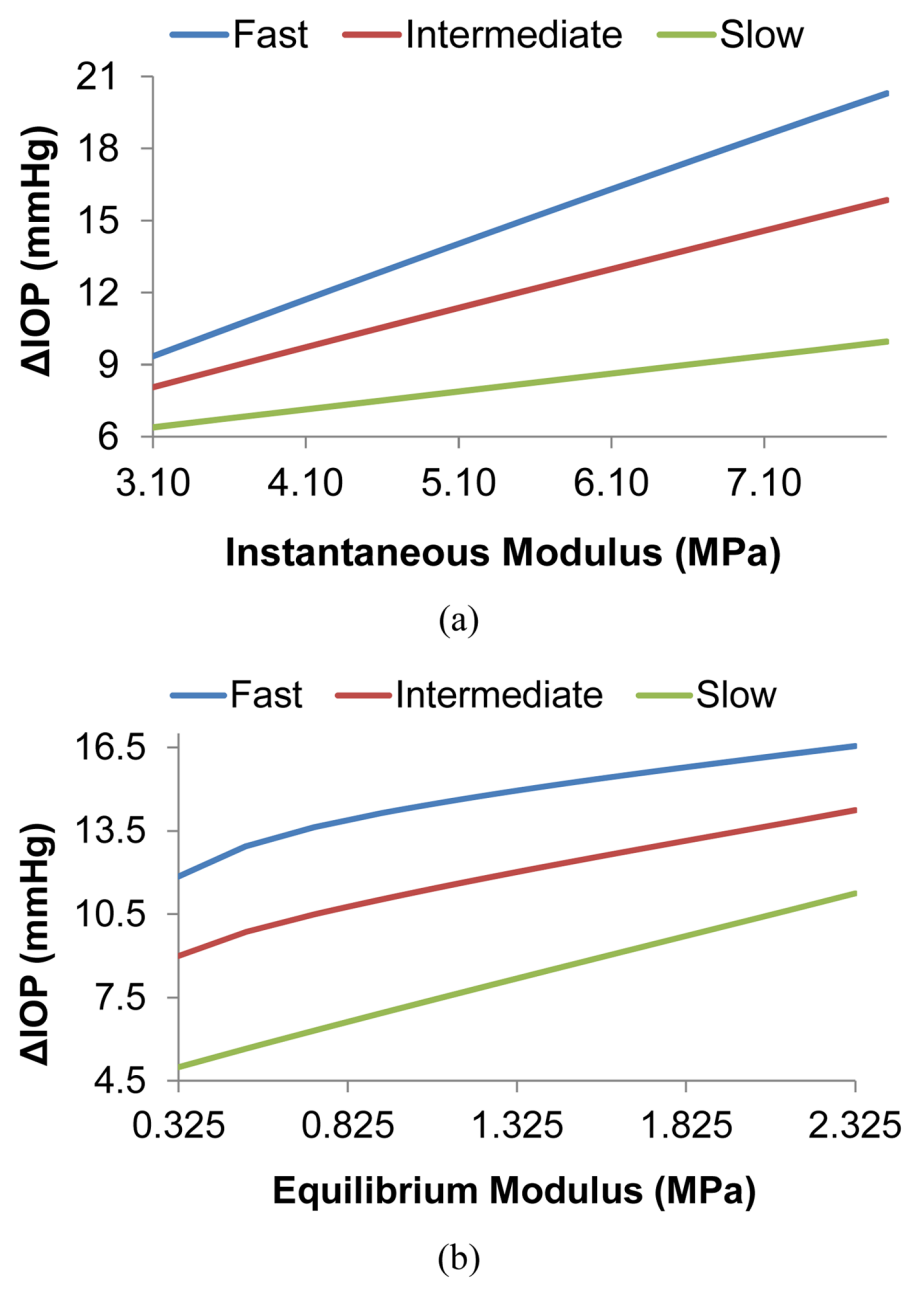
The effects of corneal and scleral biomechanical properties on IOP elevations at different infusion rates. (a) Effects of instantaneous modulus, (b) Effects of equilibrium modulus.

Figure 3 presents the effects of instantaneous modulus and equilibrium modulus on IOP elevations during micro-volumetric changes in the eye at different rates. The instantaneous moduli of the cornea and the sclera were varied proportionally (*i.e*., 1:5) while all other parameters were kept at their baseline. In general, IOP elevations were higher at higher modulus regardless of the infusion rates. Figure 3(a) shows that the IOP elevations increased almost linearly with the instantaneous moduli of the cornea and sclera at all rates. The slope however was different for different rates with a larger slope at the higher infusion rate. Figure 3(b) shows the effects of the equilibrium modulus. The response was less linear for the fast and intermediate infusion rates, particularly at the lower modulus range.

**Figure 4:**
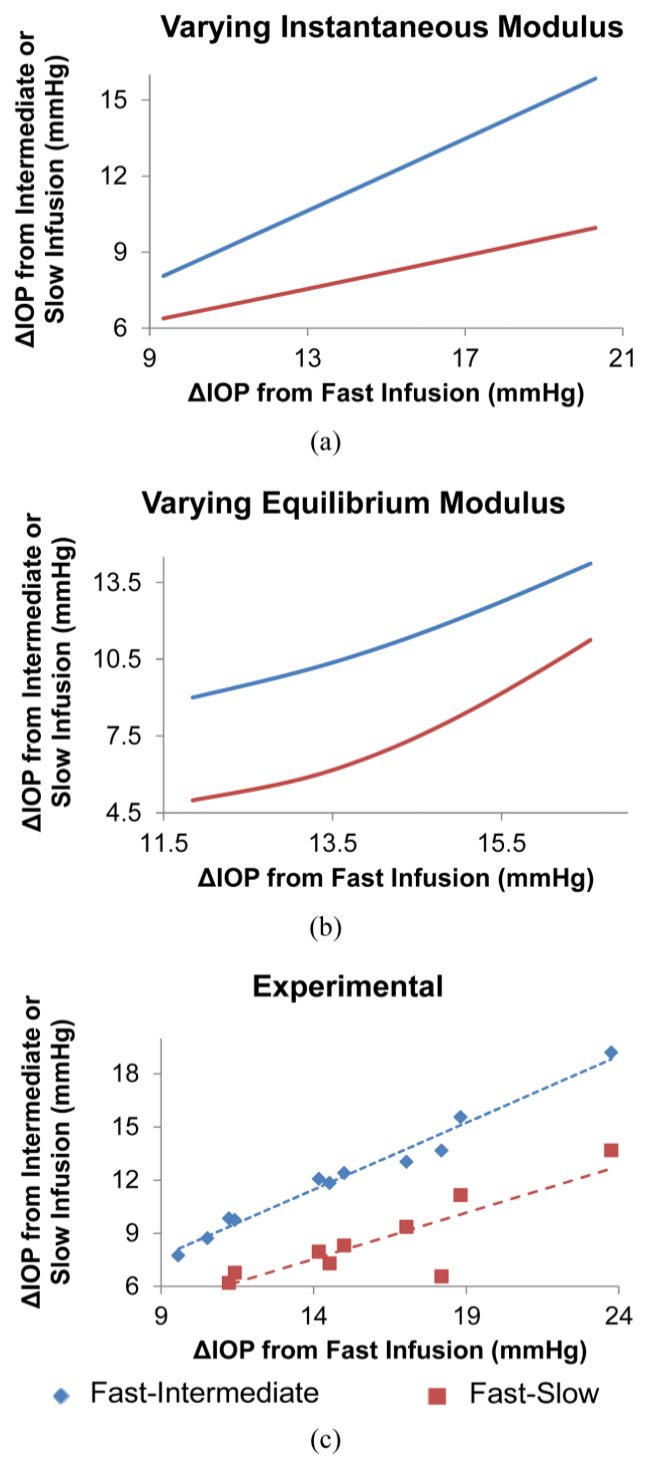
The relationships of IOP elevations at different infusion rates in: (a) A group of simulated eyes that only differ in instantaneous modulus; (b) A group of simulated eyes that only differ in equilibrium modulus; and (c) 11 porcine eyes used in the experiments [38].

Treating each data point in Figure 3(a) as an individual eye, the data points form a group of simulated eyes that only differ in instantaneous modulus. The relationships of the IOP elevations at different infusion rates in the group of eyes are plotted in Figure 4(a), showing that the IOP elevations at different infusion rates are predicted to be linearly correlated in a group of eyes that only differ in the instantaneous modulus. A similar plot for the eyes that only differ in equilibrium modulus (using data points in Figure 3(b)) is shown in Figure 4(b). The relationships of IOP elevations at different infusion rates become nonlinear for this case. Figure 4(c) shows the experimental results obtained from 11 porcine eyes, showing that the IOP elevations at different infusion rates were approximately linearly correlated in the tested eyes (R = 0.99, p < 0.001, for fast and intermediate correlation; R = 0.89, p < 0.001, for fast and slow correlation) [28].

**Figure 5:**
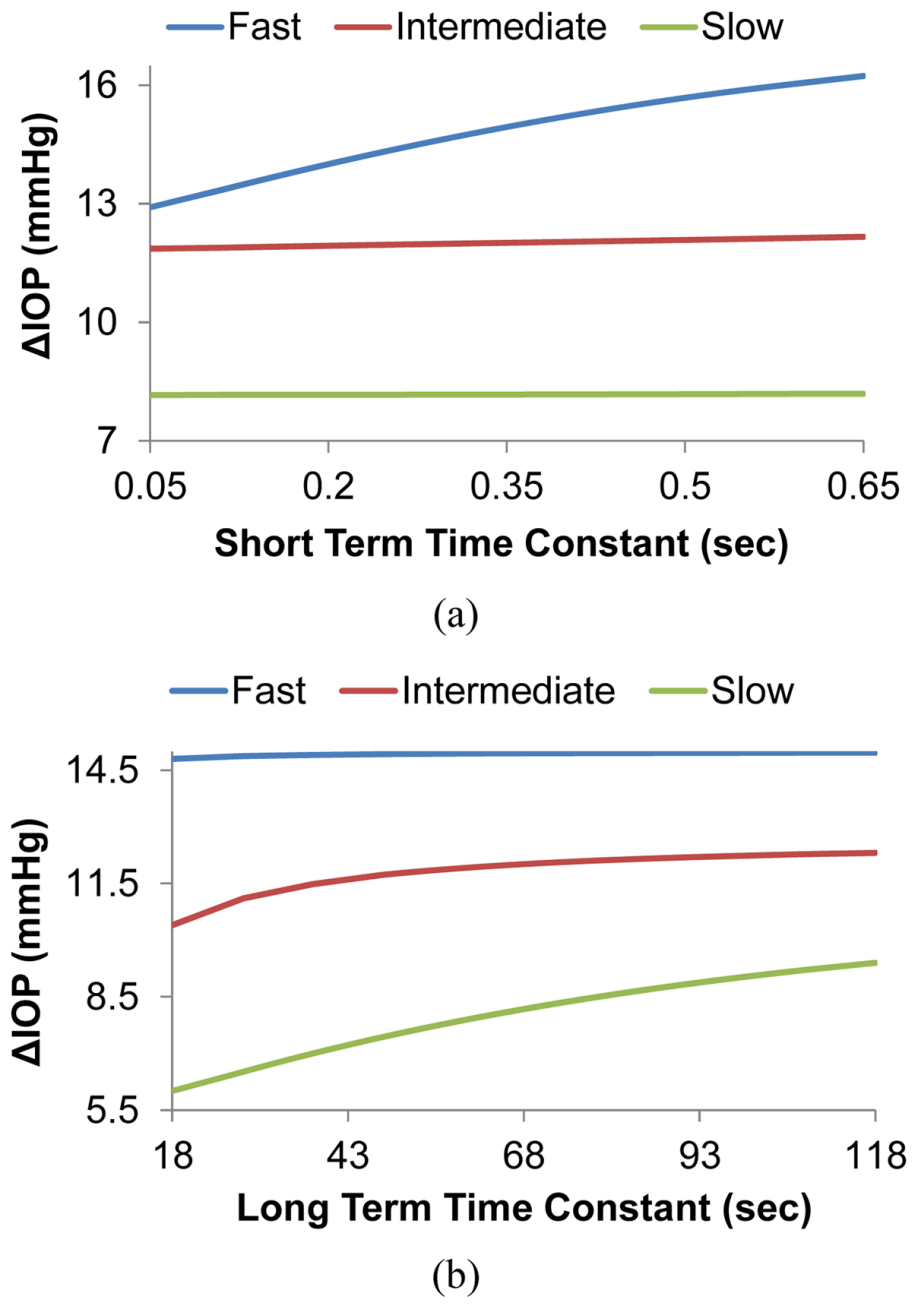
The effects of (a) the short term time constant and (b) the long term time constant on IOP elevations at different infusion rates.

Figure 5 presents the effect of the time constants on the IOP elevations due to micro-volumetric changes at different infusion rates. Figure 5(a) shows that the short term time constant affects IOP elevations during fast volumetric changes but not during intermediate or slow volumetric changes. A larger short term time constant results in a larger IOP elevation for fast volumetric changes. Conversely, the long term time constant affects the intermediate and slow volumetric changes but not the fast (Figure 5(b)). A larger long term time constant results in larger IOP elevations for the slow and intermediate rates.

**Figure 6:**
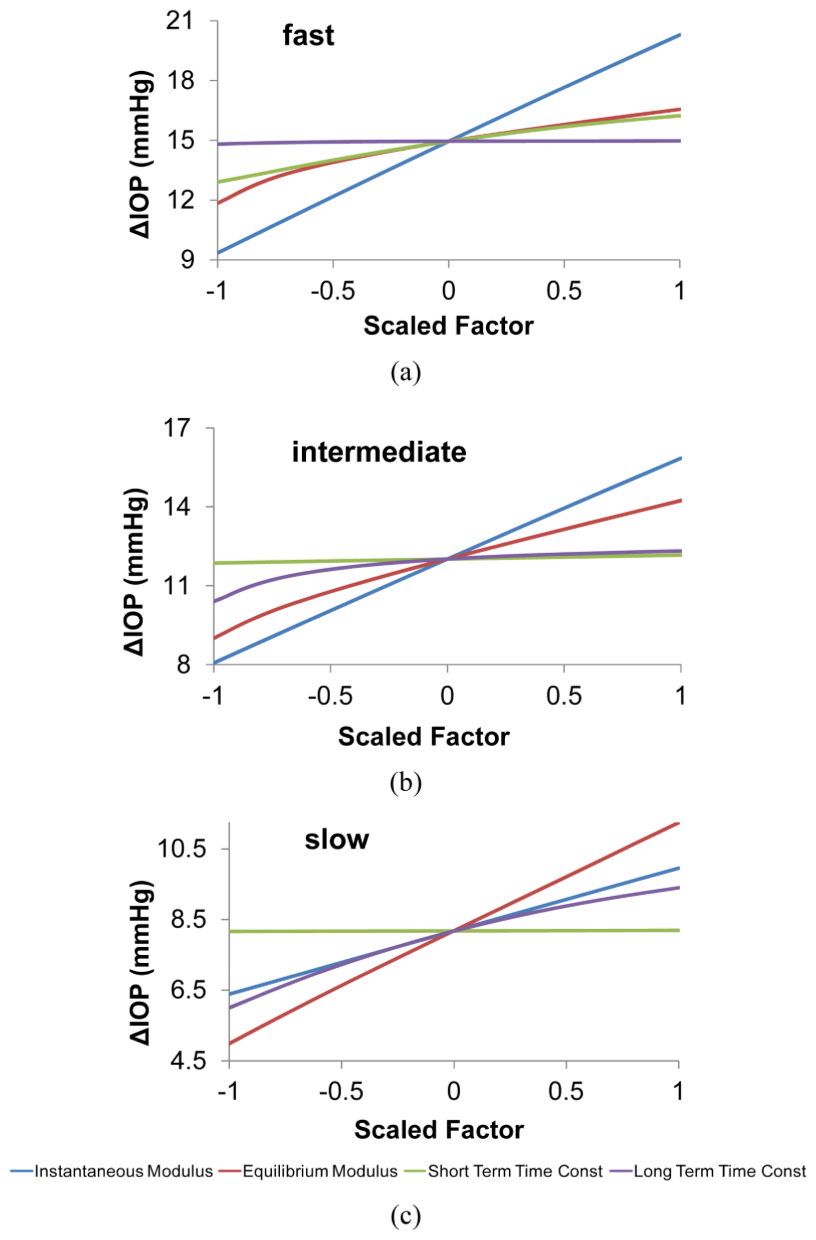
Effects of all corneal scleral factors on IOP elevations due to micro-volumetric changes at the (a) fast, (b) intermediate, and (c) slow rate.

Figure 6 shows the comparative influence of each of the corneal and viscoelastic properties on the IOP elevations, separately presented for each infusion rate. The x-axis shows the value for all parameters linearly scaled from its minimum value ( 1) to maximum value (+1). It is found that the instantaneous modulus has the largest influence for the fast and intermediate rates, but its influence diminishes for the slow infusion rate, for which the equilibrium modulus takes the dominant role. The time constants generally have smaller effects than the moduli, but their influence could be comparable to one of the two types of moduli depending on the rates.

**Figure 7:**
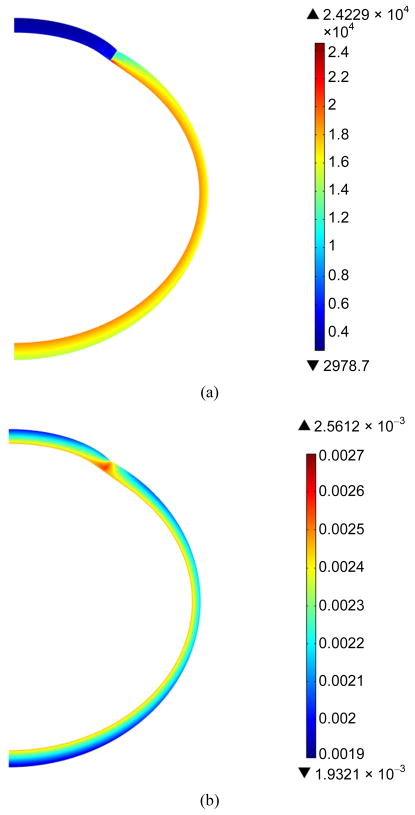
Color maps of von Mises stress (a) and first principal strain (b) in the cornea and the sclera at the end of the fast infusion (15 μL/s for 1 second).

Figure 7 shows the distribution of the von Mises stresses and principal strains in the cornea and the sclera at the end of the fast infusion. The stresses and strains were apparently different in the cornea as compared to those in the sclera, due to the discrepancy in the material properties. The stresses and strains also showed variations through the thickness as contrasted to what a thin-shell model would predict [22].

## 4. DISCUSSION

In this work, we have built a finite element model of the whole ocular shell to investigate factors influencing the short-term volume/pressure changes in the eye. We found that a simple linear viscoelastic model with two branches simulating the short-term and long-term relaxation processes could adequately reproduce the experimental data obtained from the infusion experiments performed in porcine eyes (Figure 2). Although the three different infusion rates used in this study are all within the time scale of short-term IOP fluctuations seen in the eye (*i.e*., a time scale shorter than a few minutes), there was a clear rate-dependent response suggesting that the eye responds differently to micro-volumetric changes that occur within seconds, 10’s of seconds, or minutes. The larger the rate of change within this time scale, the higher the IOP elevations observed in both experiments and simulations. This rate-dependence confirmed the viscoelastic responses of the ocular shell, as well as the relevant range for the time constants of the relaxation processes in the ocular shell (*i.e*., seconds to minutes), which were found using stress relaxation tests on tissue strips [16]. Both experiments and simulations reported a linear ΔIOP/volume relationship, which was also experimentally observed by Pierscionek *et al*. in fresh porcine eye [36].

Based on the finite element model, a higher instantaneous modulus, *i.e*., a stiffer response to a step load, would result in a larger final ΔIOP for a given infusion volume regardless of the rate of change (Figure 3(a)). A similar positive relationship between ΔIOP and equilibrium modulus was also shown (Figure 3(b)). The relationship between instantaneous modulus and ΔIOP relationship was linear (*i.e*., a constant slope) while there was a degree of nonlinearity in the relationship between equilibrium modulus and ΔIOP, although that was mostly seen in the fast rate response.

In our previous experimental study, we found a strong correlation between the IOP elevations measured at different rates across different eyes (Figure 4(c)), that is, an eye that had a larger ΔIOP at the fast rate would also have a larger ΔIOP at the slower rates. Interestingly, this correlation was strongly linear (R = 0.99 or 0.89) according to the experimental data. This linear correlation could be better predicted by the variance in instantaneous modulus (Figure 4(a)) rather than the variance in the equilibrium modulus which corresponded to a more nonlinear response (Figure 4(b)).

The short term time constant had a positive relationship with ΔIOP during fast infusions, but a flat response for intermediate and slow infusions (Figure 5(a)). This time constant represents the time scale of the fast relaxation processes in the tissue. Because of the selected range of this constant in the present study (0.05 to 0.65 sec), it is expected that it does not influence the intermediate and slow rates whose time scales were far beyond this range. The long term time constant represents the slower relaxation process. As shown in Figure 5(b), the ΔIOP in the intermediate and slow infusions were noticeably affected by this time constant, but the ΔIOP in the fast infusion had a flat response because its short duration.

As discussed above, all viscoelastic properties affected the volume/pressure response, and the relative significance of each specific parameter was rate-dependent. For example, at the faster rates (e.g., the fast and intermediate infusion rates used in the present study), the instanttaneous modulus had the largest influence on the magnitude of ΔIOP (Figures 6(a) and (b)). As the rate decreased, the equilibrium modulus started to dominate (Figure 6(c)). In addition, the short-term time constant had minimal effect on intermediate and slow infusions, but significant effect on fast infusion; while the long-term time constant showed the opposite trend.

Previous computational work has shown that the posterior sclera is a major biomechanical structure that strongly influences the mechanical states of the optic nerve head where the glaucomatous injuries occur [12]. The results from the present study showed that the viscoelastic properties of the corneoscleral shell could also influence the dynamic profile of IOP. For example, a larger instantaneous modulus or equilibrium modulus (*i.e*., a stiffer corneoscleral shell) would result in higher IOP fluctuations due to micro-volumetric changes in the eye during daily activities. Although the pathophysiolgical consequences of IOP fluctuations are not fully understood, it is generally believed that they are detrimental to the delicate tissue structures in the eye, especially when they are at high magnitudes and fast acting [37,38]. It is therefore important to understand and characterize the viscoelastic properties of the ocular shell and their relationship to the dynamic IOP.

This work has several limitations. First, the eye is actually a “leaky” shell with a pressure-dependent outflow [39]. For simplicity, this pressure-dependent outflow was not implemented in the current model. Instead, a net flow rate was used as the infusion rate. This could have introduced certain inaccuracy in the calculations of the ΔIOP; however, this error was likely small due to the short time duration of the simulated experiments. Second, the relaxation moduli for the branches in the Maxwell model were set to the same values. Although this assumption has been used in the past [14], future experimental studies as well as modeling work are needed to verify this assumption and investigate its implications. Third, a linear viscoelastic model was used and the reported nonlinearity in the stress-strain relationship of the cornea and sclera was neglected. Because the corneoscleral shell is primarily a collagenous tissue, mechanical nonlinearity is expected and has been confirmed in the past. Constitutive modeling that accounts for the nonlinear responses would thus be more desirable [40–44]. However, in the present study, the IOP was always above the baseline of 15 mmHg during the simulated infusion studies, which likely positioned the eye close to or beyond the toe region of the corneoscleral response [28]. Therefore, the linear approximation, which simplifies the model, is likely sufficient in the present study. Fourth, previous studies have shown the importance of collagen microstructure (for example, anisotropic collagen fiber alignment) in affecting the stress and strain distributions in the cornea and sclera [20,45–48]. Future work should investtigate how heterogeneous microstructural and material properties could affect the eye’s response to dynamic micro-volumetric changes.

## 5. CONCLUSION

In conclusion, this study utilized finite element models of a porcine corneoscleral shell to investigate the effects of the shell’s viscoelastic properties on the IOP elevations due to volumetric changes in the eye. The major finding was that the instantaneous and equilibrium moduli, as well as the short-term and long-term time constants, had an effect on IOP elevations, and the relative significance of the specific parameters was highly dependent on the rate of change.

## Figures and Tables

**Table 1:** The infusion rates and total duration used in the experiments and simulations.

	Fast	Intermediate	Slow
**Rate**	15 μL/s	1 μL/s	0.1 μL/s
**Duration**	1 s	15 s	150 s

**Table 2:** Material parameters used in the model simulations.

Material properties	Sclera		Cornea	

	Baseline	Range	Baseline	Range
**Instantaneous modulus**	5.5 MPa	3.1 – 7.9 MPa	1.1 MPa	0.62 – 1.6 MPa
**Equilibrium modulus**	1.325 MPa	0.325 – 2.325 MPa	0.265 MPa	0.065 – 0.465 MPa
**Short term time constant**	0.35 sec	0.05 – 0.65 sec	0.35 sec	0.05 – 0.65 sec
**Long term time constant**	68 sec	18 – 128 sec	68 sec	18 – 128 sec

## References

[1] Quigley HA (1999). Neuronal death in glaucoma. Progress in Retinal and Eye Research.

[2] Musch DC, Gillespie BW, Niziol LM, Lichter PR, Varma R (2011). Intraocular pressure control and long-term visual field loss in the collaborative initial glaucoma treatment study. Ophthalmology.

[3] Liu JHK, Zhang X, Kripke DF, Weinreb RN (2003). Twenty-four-hour intraocular pressure pattern associated with early glaucomatous changes. Investigative Ophthalmology & Visual Science.

[4] Parsley J, Powell RG, Keightley SJ, Elkington AR (1987). Postural response of intraocular pressure in chronic open-angle glaucoma following trabeculectomy. British Journal of Ophthalmology.

[5] Weinreb RN, Cook J, Friberg TR (1984). Effect of inverted body position on intraocular pressure. American Journal of Ophthalmology.

[6] Rafuse PE, Mills DW, Hooper PL, Chang TS, Wolf R (1994). Effects of Valsalva’s manoeuvre on intraocular pressure. Canadian Journal of Ophthalmology.

[7] Vieira GM, Oliveira HB, de Andrade DT, Bottaro M, Ritch R (2006). Intraocular pressure variation during weight lifting. Archives of Ophthalmology.

[8] Susanna R, Vessani RM, Sakata L, Zacarias LC, Hatanaka M (2005). The relation between intraocular pressure peak in the water drinking test and visual field progression in glaucoma. British Journal of Ophthalmology.

[9] Coleman DJ, Trokel S (1969). Direct-recorded intraocular pressure variations in a human subject. Archives of Ophthalmology.

[10] Kiuchi T, Motoyama Y, Oshika T (2006). Relationship of progression of visual field damage to postural changes in intraocular pressure in patients with normal-tension glaucoma. Ophthalmology.

[11] Schuman JS (2000). Increased intraocular pressure and visual field defects in high resistance wind instrument players. Ophthalmology.

[12] Sigal IA, Flanagan JG, Ethier CR (2005). Factors influencing optic nerve head biomechanics. Investigative Ophthalmology & Visual Science.

[13] Palko JR, Pan X, Liu J (2011). Dynamic testing of regional viscoelastic behavior of canine sclera. Experimental Eye Research.

[14] Downs JC (2005). Viscoelastic material properties of the peripapillary sclera in normal and early-glaucoma monkey eyes. Investigative Ophthalmology & Visual Science.

[15] Girard M, Suh JKF, Hart RT, Burgoyne CF, Downs JC (2007). Effects of storage time on the mechanical properties of rabbit peripapillary sclera after enucleation. Current Eye Research.

[16] Downs JC (2003). Viscoelastic characterization of peripapillary sclera: Material properties by quadrant in rabbit and monkey eyes. Journal of Biomechanical Engineering.

[17] Eilaghi A (2010). Biaxial mechanical testing of human sclera. Journal of Biomechanics.

[18] Fazio MA (2012). Regional variations in mechanical strain in the posterior human sclera. Investigative Ophthalmology & Visual Science.

[19] Coudrillier B (2012). Biomechanics of the human posterior sclera: Age- and glaucoma-related changes measured using inflation testing. Investigative Ophthalmology & Visual Science.

[20] Girard MJA, Downs JC, Bottlang M, Burgoyne CF, Suh JKF (2009). Peripapillary and posterior scleral mechanics—Part II: Experimental and inverse finite element characterization. Journal of Biomechanical Engineering.

[21] Girard MJA, Downs JC, Burgoyne CF, Suh JKF (2008). Experimental surface strain mapping of porcine peripapillary sclera due to elevations of intraocular pressure. Journal of Biomechanical Engineering.

[22] Tang J, Liu J (2012). Ultrasonic measurement of scleral cross-sectional strains during elevations of intraocular pressure: Method validation and initial results in posterior porcine sclera. Journal of Biomechanical Engineering.

[23] Nguyen T, Boyce B (2011). An inverse finite element method for determining the anisotropic properties of the cornea. Biomechanics and Modeling in Mechanobiology.

[24] Nguyen TD, Jones RE, Boyce BL (2008). A nonlinear anisotropic viscoelastic model for the tensile behavior of the corneal stroma. Journal of Biomechanical Engineering.

[25] Elsheikh A, Anderson K (2005). Comparative study of corneal strip extensometry and inflation tests. Journal of the Royal Society Interface.

[26] He X, Liu J (2009). A quantitative ultrasonic spectroscopy method for noninvasive determination of corneal biomechanical properties. Investigative Ophthalmology & Visual Science.

[27] Liu J, He X (2009). Corneal stiffness affects IOP elevation during rapid volume change in the eye. Investigative Ophthalmology & Visual Science.

[28] Morris HJ (2013). Correlation between biomechanical responses of posterior sclera and IOP elevations during micro intraocular volume change. IOVS.

[29] Sródka W (2010). Goldmann applanation tonometry— Not as good as gold. Acta of Bioengineering and Biomechanics/Wroc aw University of Technology.

[30] Anderson K, El-Sheikh A, Newson T (2004). Application of structural analysis to the mechanical behaviour of the cornea. Journal of the Royal Society Interface.

[31] Asejczyk-Widlicka M, Schachar RA, Pierscionek BK (2008). Optical coherence tomography measurements of the fresh porcine eye and response of the outer coats of the eye to volume increase. Journal of Biomedical Optics.

[32] Callister WD (2003). Materials science and engineering an introduction.

[33] Mase GT, Mase GE (2010). Continuum mechanics for engineers.

[34] Ethier CR, Johnson M, Ruberti J (2004). Ocular biomechanics and biotransport. Annual Review of Biomedical Engineering.

[35] Woo SLY, Kobayashi AS, Schlegel WA, Lawrence C (1972). Nonlinear material properties of intact cornea and sclera. Experimental Eye Research.

[36] Pierscionek BK, Asejczyk-Widlicka M, Schachar RA (2007). The effect of changing intraocular pressure on the corneal and scleral curvatures in the fresh porcine eye. The British Journal of Ophthalmology.

[37] Caprioli J, Coleman AL (2008). Intraocular pressure fluctuation: A risk factor for visual field progression at low intraocular pressures in the advanced glaucoma intervention study. Ophthalmology.

[38] Resta V, Novelli E, Vozzi G, Scarpa C, Caleo M, Ahluwalia A, Solini A, Santini E, Parisi V, Di Virgilio F, Galli-Resta L (2007). Acute retinal ganglion cell injury caused by intraocular pressure spikes is mediated by endogenous extracellular ATP. European Journal of Neuroscience.

[39] Epstein DL, Rowlette LL, Roberts BC (1999). Acto-myosin drug effects and aqueous outflow function. Investigative Ophthalmology & Visual Science.

[40] Tang H, Buehler MJ, Moran B (2009). A constitutive model of soft tissue: From nanoscale collagen to tissue continuum. Annals of Biomedical Engineering.

[41] Comninou M, Yannas IV (1976). Dependence of stress-strain nonlinearity of connective tissues on the geometry of collagen fibers. Journal of Biomechanics.

[42] Maceri F, Marino M, Vairo G (2012). An insight on multiscale tendon modeling in muscle-tendon integrated behavior. Biomechanics and Modeling in Mechanobiology.

[43] Maceri F, Marino M, Vairo G (2010). A unified multiscale mechanical model for soft collagenous tissues with regular fiber arrangement. Journal of Biomechanics.

[44] Marino M, Vairo G (2012). Stress and strain localization in stretched collagenous tissues via a multiscale modelling approach. Computer Methods in Biomechanics and Biomedical Engineering.

[45] Pinsky PM, van der Heide D, Chernyak D (2005). Computational modeling of mechanical anisotropy in the cornea and sclera. Journal of Cataract & Refractive Surgery.

[46] Girard MJA, Downs JC, Burgoyne CF, Suh JKF (2009). Peripapillary and posterior scleral mechanics—Part I: Development of an anisotropic hyperelastic constitutive model. Journal of Biomechanical Engineering.

[47] Grytz R, Sigal IA, Ruberti JW, Meschke G, Downs JC (2012). Lamina cribrosa thickening in early glaucoma predicted by a microstructure motivated growth and remodeling approach. Mechanics of Materials.

[48] Grytz R, Meschke G, Jonas J (2011). The collagen fibril architecture in the lamina cribrosa and peripapillary sclera predicted by a computational remodeling approach. Biomechanics and Modeling in Mechanobiology.

